# Buffering stress in cognitive aging: the moderating role of positive emotions on trait-level stress reactivity and negative emotions in middle-aged and older adults

**DOI:** 10.3389/fpsyt.2026.1916607

**Published:** 2026-10-29

**Authors:** Rose Lin, Maria M. Quiñones-Cordero, Kathi L. Heffner

**Affiliations:** 1 Elaine Hubbard Center for Nursing Research on Aging, School of Nursing, University of Rochester Medical Center, Rochester, NY, United States; 2 Division of Geriatrics & Aging, Department of Medicine, University of Rochester Medical Center, Rochester, NY, United States; 3 Department of Psychiatry, University of Rochester Medical Center, Rochester, NY, United States

**Keywords:** cognitive aging, emotion regulation, negative affect, positive affect, stress reactivity

## Abstract

**Introduction:**

This study examined the moderating role of positive emotion in the relationship between trait-like stress reactivity and negative emotion, and whether this moderating effect varies by cognitive performance and cognitive decline across nine years.

**Methods:**

Using data from 2,041 participants in the Midlife in the United States (MIDUS) study (mean age = 65.52 years), hierarchical regression models were conducted to predict negative emotion from trait-like stress reactivity, positive emotion, and their interaction, controlling for age, gender, years of education, depressed mood, and neuroticism. Simple slope analyses were conducted to test the direction of significant interactions. Analyses were repeated within subgroups of cognitive performance and cognitive decline across 9 years.

**Results:**

The interaction between positive emotion and trait-like stress reactivity was significant, with higher positive emotion associated with a weaker relationship between stress reactivity and negative emotion. This moderating effect was observed across low, moderate, and high cognitive performance groups; a three-way interaction further indicated that the moderating effect becomes stronger as cognitive performance increases. For cognitive decline, the moderating effect was significant only among individuals with relatively greater cognitive decline, although the three-way interaction was not significant.

**Conclusion:**

Positive emotion may buffer the relationship between trait-level stress reactivity and negative emotion across the cognitive performance range, although this moderating effect appears weaker at lower cognitive performance. Findings for cognitive decline were less conclusive, as a subgroup-specific association among participants with greater decline was not confirmed when cognitive decline was analyzed as a continuous moderator.

## Introduction

Emotional responses to stress are central to psychological health across the lifespan, and impaired stress adaptation in later life contributes substantially to the burden of depression, anxiety, and cognitive decline (1, 2). Although stress adaptation has traditionally focused on major life events (3), daily hassles can also affect long-term health through repeatedly activating emotional and physiological stress responses (4). Central to this process is trait-level stress reactivity, an individual difference characterized by heightened tension, irritability, and negative emotional responding when encountering minor hassles or reflecting on everyday demands (5). People with higher stress reactivity tend to appraise stressors as threatening and respond with more intense and frequent negative emotions (6). These emotional responses are accompanied by prolonged activation of stress-induced physiological systems, including the sympathetic nervous system and the hypothalamic-pituitary-adrenal axis (7), which can reinforce persistent negative mood states and compromise stress adaptation over time.

Older adults may be particularly vulnerable to the effects of heightened stress reactivity. Although some evidence suggests that emotion regulation generally improves with age (8), older adults are also more reactive to interpersonal tensions (4), and to repeated exposure to daily stressors (9). When an individual has higher stress reactivity, recurring minor challenges may lead to negative emotions and reduced capacity to recover. Across studies, heightened stress reactivity has been associated with elevated risk for mood disorders (10), greater cardiovascular risk (11), as well as cognitive decline and progression to Alzheimer’s disease and related diseases (12). Given these long-term impacts, identifying protective factors that buffer against the consequences of heightened stress reactivity in later life represents an important priority for supporting emotional well-being in aging research.

A growing body of work points to positive emotion as a plausible buffer against negative emotional responses to stress. The premise is that positive and negative emotions do not simply lie at the opposite ends of a continuum, but are partially independent systems that can be regulated separately (13). The Broaden-and-Build theory posits that positive emotions can expand one’s thought-action repertoire, allowing more flexible cognitive appraisals and greater access to coping strategies (14). Similarly, the Undoing Hypothesis suggests that positive emotions counteract the physiological and psychological activation triggered by stress, accelerating recovery and restoring equilibrium (15). Altogether, these theories suggest that positive emotions can serve as a protective mechanism, reducing the intensity and duration of stress responses. As such, even individuals with heightened stress reactivity may be less likely to experience sustained negative emotions if positive emotions are sustained.

While this protective role of positive emotions has been widely studied in younger, cognitively healthy adults under both acute laboratory and everyday settings (15–18), a growing body of evidence suggests that this buffering effect extends into later life. Using daily diary methods, Leger, Charles (17) researchers demonstrated that positive emotions experienced on stressful days were associated with lower same-day and next-day negative emotion in adults living in the community, with effects persisting after accounting for baseline mood. Similarly, Ong, Bergeman (6) showed that among older widowed adults, those who experienced greater positive emotions during bereavement showed faster emotional recovery from daily stress, indicating that the role of positive emotions supports resilience under stressful adversity. Our recent work was the first step towards understanding the role of positive emotion in later life [citation blinded for review], showing that middle-aged and older dementia caregivers who sustained positive emotions during acute laboratory stress exhibited a weaker link between emotional reactivity and subsequent negative mood states up to 40 minutes post-stress exposure. Notably, emerging findings suggest that this benefit may extend to everyday settings. A daily diary study by Peleg, Wallimann (19) found that savoring, an emotion regulation strategy that involves attending to positive experiences, reduced the impact of daily stress on negative mood in older adults. Cultivating positive emotional experiences may play an important role in fostering stress adaptation capacity in later life.

We suggest, however, that cognitive aging should be considered within these emotion regulatory pathways, given the overlapping neurobiological pathways underpinning emotion and cognitive functioning (20, 21). Coping strategies known to generate positive emotion under stress, such as appraisal, problem-solving coping, and positive reframing (22, 23), depend on cognitive control processes such as attentional control, set shifting, and processing speed (24, 25), all supported by prefrontal and cingulate systems involved in cognitive control of emotions (26, 27). Impairment in the same processes may also compromise the ability to disengage from negative information, as suggested by the impaired disengagement hypothesis (28), suggests that reduced attentional control may prolong rumination on negative content and lead to more intense negative emotions. Therefore, cognitive performance may moderate the extent to which positive emotions can be leveraged to counteract stress-induced negative emotion. This moderating role is particularly relevant to midlife and later life because age-related brain and neuropathological changes become more evident after the age of 50 (29–32), and even subtle age-related cognitive deficits can compromise one’s capacity for stress adaptation and emotion regulation (8). However, whether such deficits also weaken the buffering role of positive emotions remains unclear.

This gap is further complicated by the fact that cognitive performance and cognitive decline capture distinct dimensions of cognitive aging. In this study, we define cognitive performance as the level of cognitive resources available, while cognitive decline is the within-person change in cognitive function over time and may indicate an ongoing adjustment to cognitive losses. Each dimension may exert different impacts on emotion regulation, and neither of them has been examined for its correlation with the buffering role of positive emotions. Importantly, recent evidence suggests that the capacity to experience positive emotions (e.g., mean level, frequency, and intensity) remains largely preserved in people with cognitive deficits (33–35). This pattern raises the possibility that even when cognitive control resources are diminished, positive emotions may continue to exert downstream regulatory effects, potentially through more automatic or affective pathways that are less dependent on effortful cognitive engagement (36).

To date, no community-based study has directly tested whether the buffering role of positive emotions varies by level of cognitive performance or by degree of longitudinal cognitive decline. If positive emotions continue to mitigate stress responses in this context, interventions that enhance positive emotional experiences may offer a novel strategy to strengthen stress adaptation capacity in older adults and potentially delay further cognitive decline. Building upon our prior laboratory findings (37), the current study tested the regulatory role of positive emotions in a broader, community-based sample using data from the Midlife in the United States study (MIDUS) (38), a national longitudinal study of community-dwelling middle-aged and older adults’ health and well-being. In this study, we examined whether positive emotions moderate the relationship between trait-level stress reactivity and negative emotions, and whether global cognitive performance, as well as a longitudinal cognitive decline across a 9-year period, affects the strength or direction of this moderating effect.

Hypothesis 1 (H1): Higher trait-level stress reactivity is correlated with higher levels of negative emotion.

Hypothesis 2 (H2): Positive emotions significantly moderate the relationship between trait-level stress reactivity and negative emotions; those with higher levels of positive emotions exhibit a weakened association between trait-level stress reactivity and negative emotions.

Exploratory question 1: How do different levels of cognitive performance affect the moderating role of positive emotions in the relationship between trait-level stress reactivity and negative emotions?

Exploratory question 2: How do different levels of cognitive decline affect the moderating role of positive emotions in the relationship between trait-level stress reactivity and negative emotions?

## Materials and methods

The current study analyzed participants from the second (MIDUS 2; 2004-2006) and third (MIDUS 3; 2013-2014) waves of the MIDUS study, focusing on participants enrolled in the Cognitive Project Component. This component involved a comprehensive battery of cognitive testing, including standardized measures of executive functioning and memory. A detailed description of the MIDUS study is available online (http://midus.wisc.edu/scopeofstudy.php). This study was exempt from Institutional Review Board oversight (29). MIDUS datasets are publicly available (https://www.icpsr.umich.edu/sites/rcmd/view/collections/203).

### Participants

Participants included in the current analyses were limited to those aged 50 or older at the time of MIDUS 2 and who completed cognitive assessments at both MIDUS 2 and MIDUS 3. This age range was selected to correspond to the period when cognitive decline (normative or neuropathological) first becomes apparent (32). It also aligns with prior work using MIDUS data to examine cognitive trajectories across those aged 50+ (29).

Details of the data collection procedures have been reported elsewhere (39). In brief, the data collection consisted of a 30-min telephone interview including questions assessing psychological distress and mood over the past 30 days, followed by a mailed self-administered questionnaire and a second telephone interview to complete the cognitive tasks. Participants with missing data on key variables were excluded from the analyses.

### Measures

**Table 1** shows the items included in the measures. Trait-level stress reactivity was assessed using the 3-item subscale of the Multidimensional Personality Questionnaire (MPQ) (40), which captures an individual’s tendency toward heightened negative emotional responses to stressful situations and demands. Specifically, items assessed the degree to which participants reported, over the past 30 days, becoming tense in response to daily demands, experiencing rapid mood shifts when small things went wrong, and feeling irritable in the face of minor hassles. These items reflect a tendency toward heightened negative emotional responding. Participants rated each item on a 4-point Likert scale ranging from “True to you” to “False”; item scores were summed, with higher scores indicating greater stress reactivity. This subscale showed good internal consistency in the current sample (alpha = 0.729), with prior studies demonstrating good internal consistency (alpha = 0.74), convergent and discriminant validity (41–43).

**Table 1:** Items included in outcome measures.

Items included
3-item stress reactivity subscale of the Multidimensional Personality Questionnaire (item code: [C1SMPQSR]) 1. My mood often goes up and down 2. I sometimes get myself into a state of tension and turmoil as I think of the day’s events. 3. Minor setbacks sometimes irritate me too much.
5-item PANAS on negative emotions (item code: [C1SNEGPA]) 1. “afraid?” 2. “jittery?” 3. “irritable?” 4. “ashamed?” 5. “upset?”
4-item PANAS on positive emotions (item code: [C1SPOSPA] 1. “enthusiastic?” 2. “attentive?” 3. “proud?” 4. “active?”
4-item self-developed subscale on neuroticism (item code: [C1SNEURO]) 1. Moody 2. Worrying 3. Nervous 4. Calm
7-item depressed emotions (item code: [C1PDEPAF]) 1. “lose interest in most things?” 2. “feel more tired out or low on energy than is usual?” 3. “lose your appetite?” or A65a “appetite increased” 4. “have more trouble falling asleep than usual?” 5. “have a lot more trouble concentrating than usual?” 6. “feel down on yourself, no good, or worthless?” 7. “think a lot about death?”

Positive and negative emotions (affect) were measured by the Positive and Negative Affect Schedule (PANAS) (44), including a 4-item positive affect subscale (PANAS-PA; enthusiastic, attentive, proud, and active) and a 5-item negative affect subscale (PANAS-NA; afraid, jittery, irritable, ashamed, upset), respectively. Each item was rated on a 5-point Likert scale from 1 (all of the time) to 5 (none of the time) using a single retrospective report of their experiences over the previous 30 days. Both subscales demonstrated good internal consistency in the current sample (PANAS-NA: Cronbach’s α = 0.848; PANAS-PA: Cronbach’s α = 0.911). Prior literature has indicated that positive and negative emotions represent two distinct affect dimensions with low shared variance (45), and moderate negative correlation (r=-0.41) was observed between PANAS-PA and PANAS-NA in the current analysis. Therefore, the two subscale scores were treated as separate predictors to assess the moderating and dependent variables, respectively.

Cognitive performance was assessed using the Brief Test of Adult Cognition by Telephone (BTACT) (46), which includes six subtests: word list recall, digits backward, category fluency, number series, and backward counting. Composite scores were calculated by standardizing each subtest score (z-scores) and then averaging the relevant z-scores. To assess changes in cognitive performance over time, we calculated the difference in composite scores between MIDUS 2 and MIDUS 3. A negative change score indicated a decline in cognitive performance over the 9-year period.

All analyses controlled for age (years), gender, education (years), depressed mood, and neuroticism, given their known association with stress regulation and emotional reactivity (47). Neuroticism was assessed using the 4-item neuroticism subscale of the MIDUS Big Five Scale. Participants rated each item on a 4-point Likert scale from 1 (a lot) to 4 (not at all), with higher scores indicating higher levels of neuroticism. This subscale was reliable (α = 0.714) in the current sample. Depressed mood was assessed with a 7-item Depressed Mood scale. Participants answered yes/no for whether they experienced each symptom during the past 12 months; responses were summed, with higher scores indicating greater depressed mood.

### Data analysis

Analyses were conducted with IBM SPSS Statistics (version 29) (48) and PROCESS macro (49). Descriptive statistics were computed for demographic and study variables. Pearson’s correlation was used to examine relationships among the variables and covariates.

H1 and H2 were tested with hierarchical regression models using MIDUS 3 data. Independent variable (3-item MPQ emotional reactivity to daily stressors subscale) and moderating factor (PANAS-PA) were mean-centered. PANAS-NA was regressed onto the MPQ and covariates to assess the effect of stress reactivity on negative emotions (H1). PANAS-PA and its interaction with stress reactivity (MPQ) were then included in the model to examine potential moderation by PANAS-PA (H2). Significant interactions were explored through simple slope analyses, which examined the relationship between stress reactivity and negative emotions at low (1 standard deviation [SD] below the mean), mean, and high (1 SD above the mean) levels of positive emotions.

To examine the effect of cognitive performance on the moderating effect of positive emotion (Exploratory question 1), participants were divided into three subgroups based on their BTACT composite scores in MIDUS 3: having low (i.e., 1^st^ to 33^rd^ percentile), moderate (34^th^ to 66^th^ percentile), and high cognitive performance (67^th^ to 100^th^ percentile). Hierarchical regression and simple slope analyses as described above were conducted separately for each subgroup.

To examine the effect of varying degrees of cognitive decline on the moderating effect of positive emotions (Exploratory question 2), subgroup analyses were conducted among individuals who showed a decline in BTACT composite scores across the 9-year interval between MIDUS 2 and MIDUS 3. These participants were divided into three subgroups based on the degree of their cognitive decline: having low (i.e., 1st to 33rd percentile), moderate (34th to 66th percentile), and high levels of cognitive decline (67th to 100th percentile). Hierarchical regression and simple slope analyses were again conducted for each subgroup. These subgroups reflect relative differences in the magnitude of the 9-year change in cognitive performance within a community-based, non-clinically defined sample. The BTACT has no validated clinical cutoff scores, and MIDUS does not include diagnostic assessment, so tertile stratification was used to allow subgroup-specific regression and simple slope analysis.

We also explore the role of cognitive performance and cognitive decline by analyzing them as a continuous moderator. Two three-way interaction models, including stress reactivity × positive emotion × BTACT composite score, and stress reactivity × positive emotion × continuous nine-year change in BTACT composite score, were tested by the PROCESS macro (Model 3). Both models controlled for the same covariates as the primary analyses. .

## Results

**Table 2** presents the means, SD, and bivariate correlations of all study variables. The participants (n = 2041) had a mean age of 65.52 years (SD = 9.54; range 50-94) and received 7.64 years of education; nearly half of them were female (56.0%). Most participants identified as White (89.7%), followed by Black or African American (2.9%), Hispanic/Latino (2.7%), Native American (1.0%), Asian (0.4%), and Native Hawaiian or Pacific Islander (0.1%).

**Table 2:** Mean, standard deviations, and Pearson’s correlation among study variables (n = 2041).

Constructs	Mean	SD	Age (years)	Education (years)	Cognitive performance (BTACT composite score^)	Neuroticism (Big Five Personality trait)	Depressed mood	Stress reactivity (MPQ)	Positive emotions (PANAS-PA)	Negative emotions (PANAS-NA)
Age (years)	65.52	9.54	--							
Education (years)	7.64	2.50	-0.093**	--						
Cognitive performance (BTACT composite score^)	-0.02	0.67	-0.448**	0.406**	--					
Neuroticism (Big Five Personality trait)	2.02	0.61	-0.128**	-0.136**	-0.049**	--				
Depressed mood	0.45	1.54	-0.072**	-0.078**	-0.014**	0.210**	--			
Stress reactivity (MPQ)	5.78	2.15	-0.107**	-0.099**	-0.070**	0.627**	0.217**	--		
Positive emotions (PANAS-PA)	3.59	0.77	0.068**	0.086**	0.065**	-0.372**	-0.287**	-0.383**	--	
Negative emotions (PANAS-NA)	1.46	0.52	-0.103**	-0.071**	-0.053*	0.506**	0.303**	0.505**	-0.413**	--

SD, standard deviation; PANAS, Positive and Negative Affect Schedule; MPQ, Multidimensional Personality Questionnaire; PA, positive affect; NA, negative affect.

^^^BTACT score assessed at MIDUS 3.

*p **<**0.05; ** = p < 0.01.

### Hypothesis testing

As hypothesized, greater trait-level stress reactivity was significantly associated with higher levels of negative emotions after controlling for age, gender, years of education, depressed mood, and neuroticism (**Table 3**). This relationship was significantly moderated by positive emotions, as evidenced by a significant interaction between PANAS-PA and MPQ. The interaction term accounted for an additional 3.1% of the variance in negative emotions, indicating a small-to-moderate effect size. Simple slope analysis (**Figure 1**) further indicated that the positive association between stress reactivity and negative emotions was stronger among those with low positive emotions (1 SD below the mean) and slightly attenuated among those with mean and high levels of positive emotions (1 SD above the mean). In other words, among participants with similar levels of stress reactivity, those with higher positive emotions reported lower negative emotions.

**Table 3:** Results of hierarchical regression analyses predicting negative emotions (PANAS-NA) with stress reactivity (MPQ), positive emotions (PANAS-PA), and their interaction as predictors.

Steps	Predictors	All participants (n = 2041)		Lower level of cognitive performance^ (n = 680)		Moderate level of cognitive performance (n = 681)		Higher level of cognitive performance (n = 680)	
Steps and predictors		Final b	Final β	Final b	Final β	Final b	Final β	Final b	Final β
1.	Constant	0.67***		0.37		0.55***		1.15***	
	Age (years)	0.00	-0.02	0.00	-0.01	0.00	-0.03	0.00	-0.05
	Gender	0.02	0.02	0.09	0.07	-0.02	-0.02	-0.02	-0.02
	Education (years)	0.00	0.02	0.01	0.05	0.01	0.05	-0.01	-0.05
	Neuroticism	0.22***	0.26***	0.23***	0.24***	0.28***	0.35***	0.15***	0.20***
	Depressed mood	0.04***	0.13***	0.04***	0.11***	0.04***	0.11***	0.06***	0.18***
	Stress reactivity	0.13***	0.55***	0.14***	0.52***	0.15***	0.64***	0.12***	0.54***
	R^2^	0.35***		0.33***		0.38***		0.32***	
2.	Positive emotions	0.00	0.01	0.01	0.01	0.03	0.04	0.00	0.00
	Stress reactivity x positive emotions	-0.02	-0.31***	-0.02*	-0.25*	-0.03***	-0.40***	-0.02*	-0.30*
	R^2^	0.38***		0.36**		0.42***		0.35***	
	Δ R²	0.03***		0.02**		0.04***		0.03***	

B, unstandardized regression coefficient; PANAS, Positive and Negative Affect Schedule; MPQ, Multidimensional Personality Questionnaire; PA, positive affect; NA, negative affect.

a ΔR² of 0.02, 0.13, and 0.26 indicating small, moderate, and large effect sizes, respectively.

ˆBased on the BTACT score assessed at MIDUS 3.

^†^p <.10. *p <.05. **p <.01. ***p <.001.

**Figure 1:**
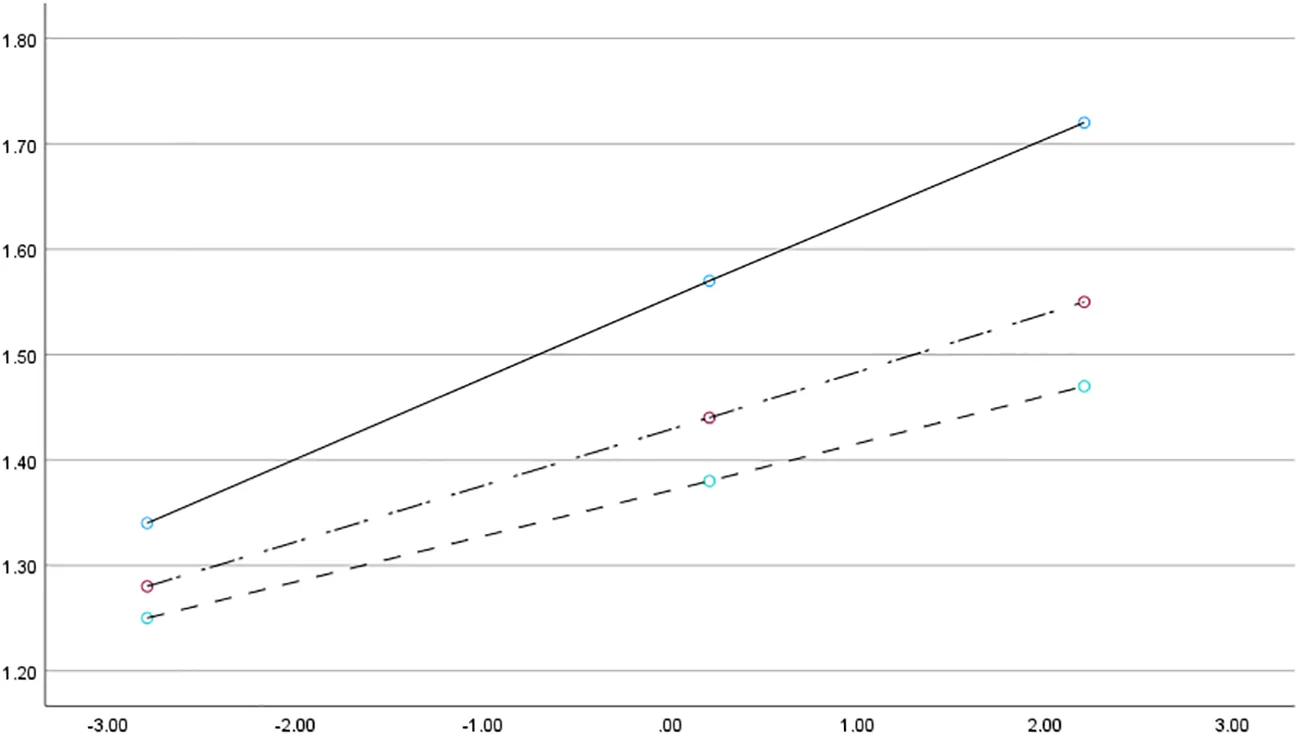
Graphic presentation of significant trait-level stress reactivity (MPQ) x positive emotions (PANAS-PA) interactions predicting negative emotions (PANAS-NA). The lines represent the association between trait-like stress reactivity and negative emotions at low (-1 SD), mean, and high (+1 SD) levels of positive emotions. Analyses were adjusted for age, gender, education, depressed mood and neuroticism (n = 2041).

### Exploratory analyses

To explore the effects of cognitive performance on the role of positive emotions, participants were categorized into three subgroups based on their BTACT composite scores: low (n = 680), moderate (n = 681), and high (n = 680) cognitive performance. Participants in the low cognitive performance group showed significantly higher stress reactivity (p < 0.01) and lower positive emotions (p = 0.02), compared to those in higher cognitive groups (**Supplementary Table S1**).

Separate hierarchical regression analyses were conducted for each cognitive performance subgroup. Across the three subgroups, stress reactivity was positively associated with negative emotions (**Table 3**). The interaction between stress reactivity and positive emotions was statistically significant in each group. Simple slope analyses also showed a consistent pattern across subgroups (**Supplementary Figures 1–3**); among those with low levels of positive emotions (1 SD below the mean), stress reactivity was associated with higher negative emotions. This association weakened as positive emotions increased to mean and high level (1 SD above the mean). These findings suggested that, regardless of cognitive performance levels, participants with greater positive emotions showed lower negative emotions even when experiencing similar stress reactivity.

Further, participants were grouped into low (n = 375), moderate (n = 376), and high (n = 376) levels of cognitive decline based on changes in BTACT composite scores over a 9-year period (**Supplementary Tables S2**, **3**). The interaction between stress reactivity and positive emotions was significant only among participants with greater cognitive decline, suggesting that positive emotions moderate the relationship between stress reactivity and negative emotions. This interaction was not significant in the low or moderate decline group. Simple slope analyses further indicated that, among participants with greater cognitive decline, the association between stress reactivity and negative emotions weakened as the level of positive emotions increased from low to high (**Supplementary Figure 4**).

### Analyses using continuous cognitive moderators

For cognitive performance (**Supplementary Table S4**), the three-way interaction of stress reactivity x positive emotion x cognitive performance was significant (b = -.02, SE = .01, p = .01), and the stress reactivity × positive emotion interaction was strongest at high cognitive performance, significant among those at the mean, and was weaker and marginally significant at low levels (p=0.05). For cognitive decline (**Supplementary Table S5**), the three-way interaction was not significant (b = .02, SE = .01, p = .15). Therefore, although the subgroup analysis identified a significant moderating effect within the greater-decline subgroup, analysis using cognitive decline as a continuous moderator did not support such finding.

## Discussion

Our findings supported H1, showing that higher trait-level stress reactivity was associated with higher negative emotions. This pattern aligns with stress process models in which individual differences in stress reactivity shape how burdensome routine demands are experienced and, in turn, increase negative emotion. Our results also supported H2 by showing that positive emotions moderate this association, and this effect was evident across cognitive performance levels (Exploratory Question 1). In our stratified analyses, individuals with lower cognitive performance reported significantly higher stress reactivity, whereas mean negative emotion did not differ across cognitive groups. The Positive emotion × stress reactivity interaction remained significant within each group, suggesting that there is no cognitive threshold at which this protective effect may be lost. Analyses using continuous cognitive moderators further suggested that this moderating effect is stronger among those with better cognitive performance and was marginally significant among those with lower cognitive performance, suggesting that the protective role may be weaker, but not absent at a lower level of cognitive functioning.

We found that stress reactivity correlated with neuroticism (r=0.63) and negative emotions (r=0.51), and that positive and negative emotions were correlated (r=-0.41), raising the question of whether these associations reflect shared measurement variance rather than distinct constructs. This concern is directly addressed by our analysis design, where neuroticism was entered as a covariate in all analyses, and the moderation effect remained significant, suggesting that the findings are not attributable to variance shared with trait negative emotionality. Prior validation studies also support the convergent and discriminant validity of the MPQ stress reactivity subscale against related measures of negative emotions (41–43). The correlation between positive and negative emotions also indicated that they do not lie on a single bipolar dimension, and aligns with evidence that positive and negative affect are regulated by partially independent pathways (13).

Our findings indicated that those with lower cognitive performance show relatively greater stress reactivity. Although our analysis relied on a global cognitive index and could not isolate specific cognitive domains, it is plausible that reduced executive function may play a key role in compromising effective stress regulation (50–52). Executive processes such as inhibitory control, cognitive flexibility, and attentional shifting support disengagement from stressors, stress appraisal, and the implementation of coping strategies (52), and poorer executive function in older adults has been linked to less flexible strategy use, greater difficulty in reappraisal, and higher perceived stress (52, 53). For those experiencing cognitive decline, repeated challenges in everyday tasks that require planning and monitoring, such as keeping track of appointments or locating misplaced items, may lead to irritability and tension, which are captured by the MPQ stress reactivity (54). To identify more specific pathways through which cognitive decline affects stress regulation, future research should use more targeted assessments of executive functions and combine these with ecological momentary assessment to capture how executive demands and positive emotions shape stress responses in daily life, an approach already shown to be feasible among people with mild cognitive impairment (55–57).

To our knowledge, this is the first study that examines the role of positive emotions across varying levels of cognitive performance in middle-aged and older adults. The buffering effect of positive emotions remained evident among those with lower cognitive performance. These findings extend prior work suggesting that both the experience of positive emotions and its stress-buffering function may be preserved in older adults undergoing cognitive change. One possible explanation is that positive emotion operates as a compensatory pathway to support stress adaptation even when cognitive resources are limited. This view is consistent with the Broad-and-Build theory that conceptualizes positive emotions as expanding momentary thought–action repertoires and building enduring regulatory capacities (14, 58, 59). It is also supported by experimental work showing that induced positive mood and discrete positive emotions (e.g., joy or gratitude) enhance task-switching performance by facilitating disengagement from no longer relevant negative information (60, 61). Neuroimaging studies further suggested that positive emotion modulates shared prefrontal control systems involved in both cognitive flexibility and emotion regulation (62, 63). These findings highlighted the potential value of interventions that cultivate positive emotions as complementary strategies alongside cognitive training [e.g., integrating mindfulness cognitive training (64)].

When we examined the nine-year change in cognition (Exploratory Question 2), the moderating role of positive emotions was observed only among individuals with greater cognitive decline, not among those with lower or relatively moderate decline. However, findings from the analyses using continuous cognitive moderators indicated that the three-way interaction was not significant. It suggests that the strength of the moderating effect did not vary linearly across the level of cognitive changes. As such, the findings from the subgroup analysis should be interpreted as exploratory rather than as evidence of a definite threshold. Future study should examine this interaction in a clinical population using repeated assessments with cognitive, stress reactivity and affect.

Our analysis relied on cross-sectional self-report surveys, which offer limited insight into how buffering effects are exhibited in real time. The next step could answer similar research questions using the MIDUS daily dairy data. To strengthen ecological validity, future work should adopt ecological momentary assessment to monitor stressor exposure, affect, and context repeatedly throughout the day. This approach would allow estimation of within-person reactivity and recovery, adjustment for stressor frequency/severity, and direct tests of whether positive emotion attenuates the link between the stressor and negative emotions in everyday life (65).

## Limitations

First, although theoretical models suggest distinct regulatory systems for positive and negative emotions (13), a moderate negative correlation was observed between PANAS-PA and PANAS-NA in this study. Caution should be taken when interpreting their effects. Second, the cross-sectional, self-report design limits causal inference and offers little insight into role of positive emotions in real time. Third, all psychological constructs were assessed by self-report, and the stress reactivity measure reflects dispositional emotional responding rather than objective stressor exposure; reactivity and exposure cannot be disentangled in these data. Third, our operationalization of cognitive decline reflects a relative difference in nine-year change in cognitive performance within a non-clinical, community-based sample. These subgroups should not be interpreted as clinically significant impairment. Future work using diagnostic staging is needed to link these patterns to clinical populations. Lastly, the sample is predominantly White and well-educated. Caution should be taken when generalizing to other populations.

## Conclusions

Our findings indicate that positive emotions buffer the relationship between stress reactivity and negative emotions across low, moderate, and high levels of cognitive performance. These findings indicated that the regulatory benefits remain observable, although relatively weaker among those with lower cognitive performance. In contrast, over nine years, the moderating effect of positive emotions emerged only among individuals with greater cognitive decline, but this pattern was not confirmed by the three-way interaction analysis. Future studies using diagnostic staging and intensive longitudinal designs are needed to determine how this protective effect operates in older adults experiencing cognitive decline.

## Data Availability

Publicly available datasets were analyzed in this study. This data can be found here: https://www.icpsr.umich.edu/sites/nacda/midus.
