# Supplementary material for: Buffering stress in cognitive aging: the moderating role of positive emotions on trait-level stress reactivity and negative emotions in middle-aged and older adults
Source: Front Psychiatry. 2026 Oct 29;17:1916607. doi: 10.3389/fpsyt.2026.1916607 (PMC13754887; doi:10.3389/fpsyt.2026.1916607)
Supplement: Supplementary file 1 [file Supplementaryfile1.docx]

# Supplementary Table S1. Mean and standard deviations of negative emotions, positive emotions, and trait-level stress reactivity across participants with low, moderate, and high levels of cognitive performance (n = 2041).

|  | Low level of  cognitive performance (n = 680)  Mean (SD) | Moderate level of  cognitive performance (n = 681)  Mean (SD) | High level of  cognitive performance (n = 680)  Mean (SD) | *p*-value |
| --- | --- | --- | --- | --- |
| Cognitive ability (BTACT at MIDUS 3) | -0.76 (0.33) | -0.02 (0.17) | 0.73 (0.34) | <0.01 |
| Trait-level stress reactivity (MPQ) | 6.03 (2.23) | 5.66 (2.10) | 5.66 (2.12) | <0.01 |
| Negative emotions (PANAS-NA) | 1.49 (0.59) | 1.46 (0.48) | 1.44 (0.47) | 0.16 |
| Positive emotions (PANAS-PA) | 3.53 (0.82) | 3.59. (0.75) | 3.64 (0.72) | 0.02 |
| *Note.* PANAS = Positive and Negative Affect Schedule; MPQ, Multidimensional Personality Questionnaire; PA, positive affect; NA, negative affect. p-value indicate group differences by ANOVA. | | | | |

# Supplementary Figure 1. Graphic presentation of significant trait-level stress reactivity (MPQ) x positive emotions (PANAS-PA) interactions predicting negative emotions (PANAS-NA). The lines represent the association between trait-level stress reactivity and negative emotion at low (-1SD), mean, and high (+1SD) levels of positive emotions scores (controlled for age, gender, years of education, depressed mood, neuroticism) among participants with low levels of cognitive performance (n = 375)

Trait-level stress reactivity (MPQ)

Negative emotions (PANAS-NA)

# Supplementary Figure 2. Graphic presentation of significant trait-level stress reactivity (MPQ) x positive emotions (PANAS-PA) interactions predicting negative emotions (PANAS-NA). The lines represent the association between trait-level stress reactivity and negative affect at low (-1SD), mean, and high (+1SD) levels of positive emotions scores (controlled for age, gender, education, depressed mood, neuroticism) among participants with a moderate level of cognitive performance (n=376)

Trait-level stress reactivity (MPQ)

Negative emotions (PANAS-NA)

# Supplementary Figure 3. Graphic presentation of significant trait-level stress reactivity (MPQ) x positive emotions (PANAS-PA) interactions predicting negative emotions (PANAS-NA). The lines represent the association between trait-level stress reactivity and negative affect at low (-1SD), mean, and high (+1SD) levels of positive emotions score (controlled for age, gender, education, depressed mood, neuroticism) among participants with a high level of cognitive performance (n=376)

Trait-level stress reactivity (MPQ)

Negative emotions (PANAS-NA)

|  | Low level of cognitive decline  (n = 375)  Mean (SD) | Moderate level of cognitive decline (n = 376)  Mean (SD) | High level of cognitive decline  (n = 376)  Mean (SD) | *p-*value |
| --- | --- | --- | --- | --- |
| **Level of cognitive decline**  **(Change in BTACT scores from MIDUS 2 to MIDUS 3)** | -0.15 (0.09) | -0.47 (0.10) | -1.01 (0.33) | <0.01 |
| **Trait-level stress reactivity (MPQ)** | 5.85 (2.19) | 5.84 (2.06) | 5.74 (2.15) | 0.45 |
| **Negative emotions**  **(PANAS-NA)** | 1.46 (0.47) | 1.43 (0.48) | 1.45 (0.49) | 0.68 |
| **Positive emotions**  **(PANAS-PA)** | 3.60 (0.73) | 3.65 (0.72) | 3.58 (0.80) | 0.41 |
| Note. PANAS = Positive and Negative Affect Schedule; MPQ, Multidimensional Personality Questionnaire**;** PA**,** positive affect; NA**,** negative affect | | | | |

# **Supplementary Table S2. Mean and standard deviations of negative emotions, positive emotions, and trait-level stress reactivity across participants** with low, moderate, and high levels of cognitive decline (n = 1127)

# Supplementary Table S3. Results of hierarchical regression analyses predicting negative emotions (PANAS-NA) with Trait-level stress reactivity, positive emotions (PANAS-PA), and trait-level stress reactivity x positive emotions as predictors based on cognitive decline (n = 1127)

|  | | Low level of  cognitive decline  (n = 375) | | Moderate level of  cognitive decline  (n = 376) | | Higher level of  cognitive decline  (n = 376) | |
| --- | --- | --- | --- | --- | --- | --- | --- |
| Steps and predictors | | Final b | Final β | Final b | Final β | Final b | Final β |
| 1. | Constant | .93** |  | .70 |  | 1.06*** |  |
|  | Age (years) | .00 | -.05 | .00 | -.06 | .00 | -.06 |
|  | Gender | .02 | .02 | -.03 | -.03 | -.01 | -.01 |
|  | Education (years) | .01 | .05 | .00 | .01 | .00 | -.02 |
|  | Neuroticism | .05*** | .16*** | .03 | .09 | .03 | .10 |
|  | Depressed mood | .27*** | .34*** | .20*** | .26*** | .15** | .19** |
|  | Trait-level stress reactivity | .07 | .31 | .15*** | .62*** | .14*** | .63*** |
|  | R^2^ | 0.40 |  | 0.37 |  | 0.33*** |  |
| 2. | Positive emotions | -.06 | -.10 | .03 | .05 | .00 | .00 |
|  | Trait-level stress reactivity x positive emotions | -.01 | -.11 | -.02 | -.30 | -.03** | -.37** |
|  | R^2^ | 0.42 |  | 0.38 |  | 0.39*** |  |
|  | Δ R² | 0.02 |  | 0.01 |  | 0.06*** |  |
| Note. b = unstandardized regression coefficient; PANAS = Positive and Negative Affect Schedule; MPQ, Multidimensional Personality Questionnaire; PA, positive affect; NA, negative affect  ^a^ ΔR² of 0.02, 0.13, and 0.26 indicating small, moderate, and large effect sizes, respectively  †p < .10. *p < .05. **p < .01. ***p < .001 | | | | | | | |

# Supplementary Figure 4. Graphic presentation of significant trait-level stress reactivity (MPQ) x positive emotions (PANAS-PA) interactions predicting negative emotions (PANAS-NA). The lines represent the association between Trait-level stress reactivity and negative affect at low (-1 SD), mean, and high (+1 SD) levels of positive emotions (controlled for age, gender, education, depressed mood, neuroticism) among high cognitive decline group (n = 376).

High level of positive emotions
(1 SD above mean)
(b = 0.04, p =0.03)

Mean positive emotions
(b = 0.05, p <0.01)

Trait-level stress reactivity (MPQ)

Negative emotions (PANAS-NA)

Low level of positive emotions
(1 SD below mean)
(b = 0.07.; p <0.01)

# Supplementary Table S4. Results of sensitivity analysis of a three-way interaction among stress reactivity (MPQ), positive emotions (PANAS-PA), and cognitive performance (BTACT composite score) predicting negative emotions (PANAS-NA).

| **Predictor** | ***b*** | **SE** | ***t*** | ***p*** | **95% CI** |
| --- | --- | --- | --- | --- | --- |
| Constant | 1.06 | .09 | 11.38 | < .001 | [.87, 1.24] |
| Age (years) | -.00 | .00 | -1.98 | .05 | [-.00, .00] |
| Gender | .02 | .02 | .87 | .39 | [-.02, .05] |
| Education (years) | .01 | .00 | 1.56 | .12 | [-.00, .01] |
| Neuroticism | .22 | .02 | 11.09 | < .001 | [.18, .26] |
| Depressed mood | .04 | .01 | 6.83 | < .001 | [.03, .06] |
| Trait-like stress reactivity (SR) | .06 | .01 | 9.87 | < .001 | [.04, .07] |
| Positive emotions (PE) | -.12 | .01 | -8.87 | < .001 | [-.15, -.09] |
| Cognitive ability (BTACT) | -.04 | .02 | -2.22 | .03 | [-.07, -.00] |
| SR × PE | -.02 | .01 | -4.45 | < .001 | [-.03, -.01] |
| SR × BTACT | -.02 | .01 | -2.46 | .01 | [-.03, -.00] |
| PE × BTACT | -.01 | .02 | -.65 | .52 | [-.05, .03] |
| SR × PE × BTACT | -.02 | .01 | -2.52 | .01 | [-.03, -.00] |

**Model summary:** R = .62, R² = .38, MSE = .17, F(12, 2022) = 104.09, p < .001.

**Results of different stress reactivity × positive emotions interaction at different levels of cognitive performance**

| **Cognitive ability (BTACT)** | **Interaction effect** | ***F*** | ***p-value*** |
| --- | --- | --- | --- |
| Low (-1 SD) | -.01 | 3.74 | .05 |
| Mean | -.02 | 19.77 | < .001 |
| High (+1 SD) | -.04 | 21.20 | < .001 |

Note. b = unstandardized regression coefficient; SE = standard error; CI = confidence interval; SR = trait-level stress reactivity; PE = positive emotions; BTACT = Brief Test of Adult Cognition by Telephone composite score; PANAS = Positive and Negative Affect Schedule.

# Supplementary Table S5. Results of sensitivity analysis of a three-way interaction among stress reactivity (MPQ), positive emotions (PANAS-PA), and cognitive change (change of BTACT composite score across 9 years) predicting negative emotions (PANAS-NA).

| **Predictor** | ***b*** | **SE** | ***t*** | ***p-value*** | **95% CI** |
| --- | --- | --- | --- | --- | --- |
| Constant | 1.18 | .11 | 10.44 | < .001 | [.96, 1.40] |
| Age (years) | -.00 | .00 | -2.56 | .01 | [-.01, -.00] |
| Gender | -.01 | .02 | -.28 | .78 | [-.05, .04] |
| Education (years) | .00 | .00 | .77 | .44 | [-.01, .01] |
| Neuroticism | .21 | .03 | 8.53 | < .001 | [.16, .26] |
| Depressed mood | .04 | .01 | 4.44 | < .001 | [.02, .05] |
| Trait-like stress reactivity (SR) | .06 | .01 | 7.70 | < .001 | [.04, .07] |
| Positive emotions (PE) | -.11 | .02 | -6.33 | < .001 | [-.14, -.07] |
| Cognitive decline (ChangeBT) | .02 | .03 | .51 | .61 | [-.04, .07] |
| SR × PE | -.02 | .01 | -2.24 | .03 | [-.03, -.00] |
| SR × ChangeBT | .01 | .01 | .59 | .56 | [-.02, .04] |
| PE × ChangeBT | .03 | .04 | .75 | .45 | [-.05, .11] |
| SR × PE × ChangeBT | .02 | .01 | 1.43 | .15 | [-.01, .05] |

**Model summary:** R = .63, R² = .39, MSE = .14, F(12, 1112) = 59.79, p < .001.

**Test of highest-order unconditional interaction (SR × PE × ChangeBT):** ΔR² = .00, p = 0.15.

Note. b = unstandardized regression coefficient; SE = standard error; CI = confidence interval; SR = trait-level stress reactivity; PE = positive emotions; ChangeBT = 9-year change in BTACT composite score; PANAS = Positive and Negative Affect Schedule.
